# Crystal structure of (*E*)-4-hy­droxy-6-methyl-3-{1-[2-(4-nitro­phen­yl)hydrazinyl­idene]eth­yl}-2*H*-pyran-2-one

**DOI:** 10.1107/S2056989017000639

**Published:** 2017-01-20

**Authors:** Kirandeep, Ahmad Husain, Pooja Negi, Girijesh Kumar, Ramesh Kataria

**Affiliations:** a Department of Chemistry, Centre for Advanced Studies in Chemistry, Panjab University, Chandigarh 160 014, India; b Department of Chemistry, DAV University Jalandhar, Jalandhar 144 001, Punjab, India

**Keywords:** crystal structure, HMNP, de­hydro­acetic acid, hydrogen bonding, thermal stability

## Abstract

The title compound, C_14_H_13_N_3_O_5_ (HMNP), was synthesized by the simple condensation of *p*-nitro­phenyl­hydrazine with de­hydro­acetic acid (DHA) in a 1:1 molar ratio in ethanol. HMNP has been characterized by using FT–IR, ^1^H and ^13^C NMR and UV–Vis spectroscopic and single-crystal X-ray diffraction techniques. The crystal packing reveals strong hydrogen bonds between the NH group and the carbonyl O atom of di­hydro­pyran­one moiety, forming chains along [101]. The thermal stability of the synthesized compound was confirmed by thermogravimetric analysis and it was found to be stable up to 513 K. The UV–Vis spectrum shows the presence of a strong band at λ_max_ 394 nm. ^1^H NMR and single-crystal X-ray analyses confirmed the presence of the *enol* form of the ligand and dominance over the *keto* form. The crystal studied was a non-merohedral twin with the refined ratio of the twin components being 0.3720 (19):0.6280 (19).

## Abstract 2

(*E*)-4-Hy­droxy-6-methyl-3-{1-[2-(4-nitro­phen­yl)hydrazinyl­idene]eth­yl}-2-*H*-pyran-2-one has been synthesized and characterized by single-crystal X-ray diffraction and by using FT–IR, ^1^H and ^13^C NMR and UV–Vis spectroscopic techniques.

## Chemical context   

For the last several decades, Schiff bases have remained an important and popular area of research for the scientific community due to their simple synthesis, versatility and extensive range of applications (Cozzi, 2004 ▸; Chen *et al.*, 2008 ▸). A number of carbonyl compounds and amines have been utilized for the synthesis of Schiff bases (Zheng *et al.*, 2009 ▸; Hussain *et al.*, 2014 ▸). However, there are only a few reports where de­hydro­acetic acid (DHA) has been used for the preparation of Schiff bases for various applications (Liu *et al.*, 1991 ▸; Luo *et al.*, 1995 ▸). In some cases, DHA-based Schiff bases are used for the synthesis of metal complexes, leading to their utilization in various biomedical applications due to their anti­fungal, anti­bacterial, anti­malarial and anti­cancer activities (Chan & Wong, 1995 ▸; Erkkila *et al.*, 1999 ▸; Ganjali *et al.*, 2007 ▸; Gupta & Sutar, 2008 ▸). In general, the compounds are formed *via* a condensation product of hydrazine and the respective aldehyde or ketone in a 1:1 molar ratio. Structurally, a Schiff base (also known as an imine or azomethine) is a nitro­gen analogue of an aldehyde or ketone in which the carbonyl group (C=O) has been replaced by an imine or azomethine group.

The reaction between *p*-nitro­phenyl­hydrazine and de­hydro­acetic acid (DHA) in a 1:1 molar ratio in distilled ethanol afforded the title compound within 4 h. We report herein on its characterization by FT–IR, ^1^H and ^13^C NMR and UV–Vis spectroscopic and single-crystal X-ray diffraction techniques.
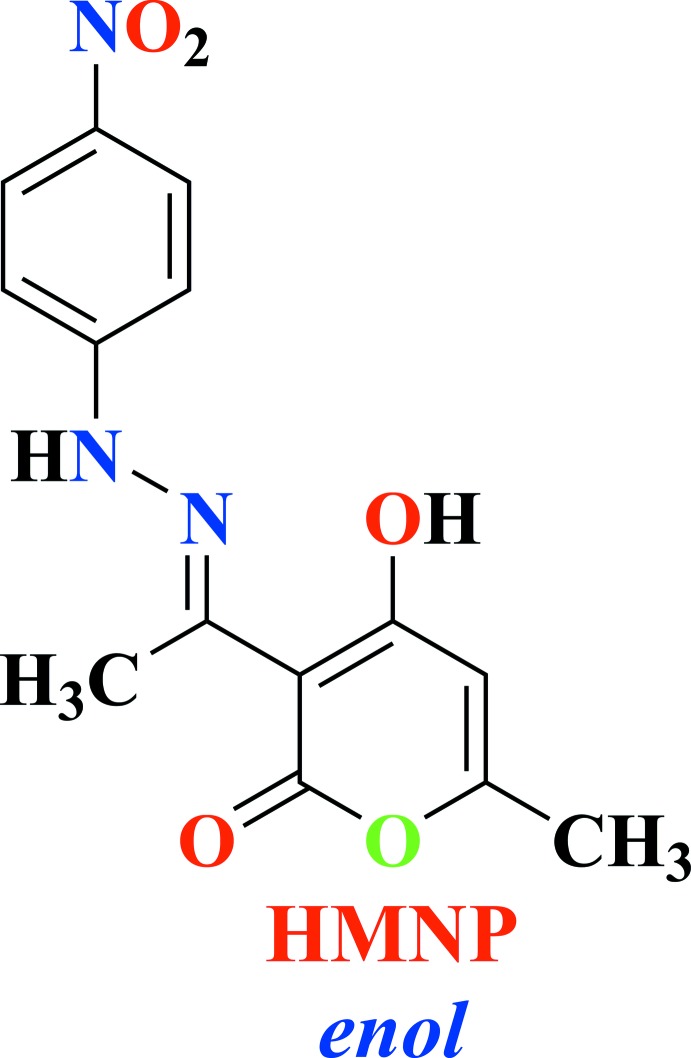


## Structural commentary   

**Figure 1:**
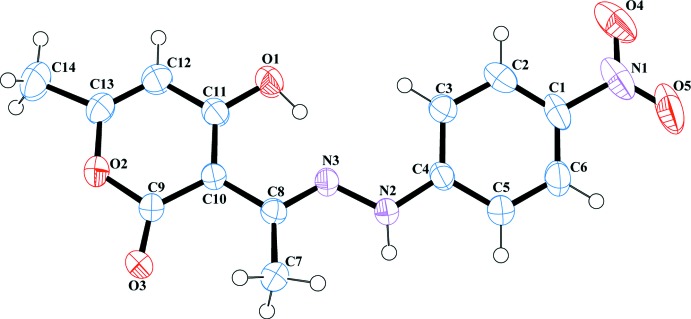
The mol­ecular structure of the title compound, showing the atom-naming scheme. The displacement ellipsoids are shown at the 50% probability level.

The mol­ecular structure of the title compound is shown in Fig. 1 ▸. The dihedral angle between the pyran (O2/C9–C13) and benzene (C1–C6) rings is 12.9 (1)°. The approximate planarity of the entire mol­ecule maybe influenced by an intra­molecular O1—H1⋯N3 hydrogen bond, which forms an *S*(6) ring.

## Supra­molecular features   

**Figure 2:**
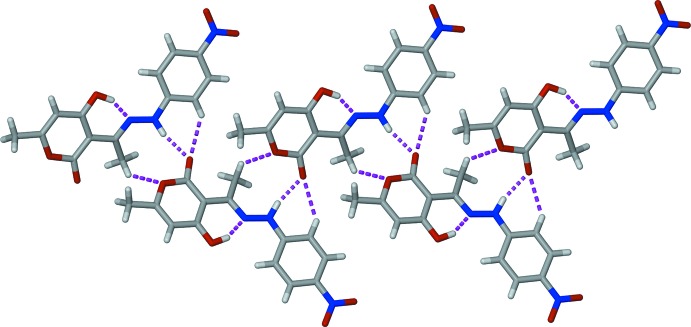
A chain parallel to [101] formed by the inter­molecular hydrogen bonding (dashed lines) between the N—H group and carbonyl O atom of the DHA moiety. Weak C—H⋯O hydrogen bonds are also shown as dashed lines.

**Figure 3:**
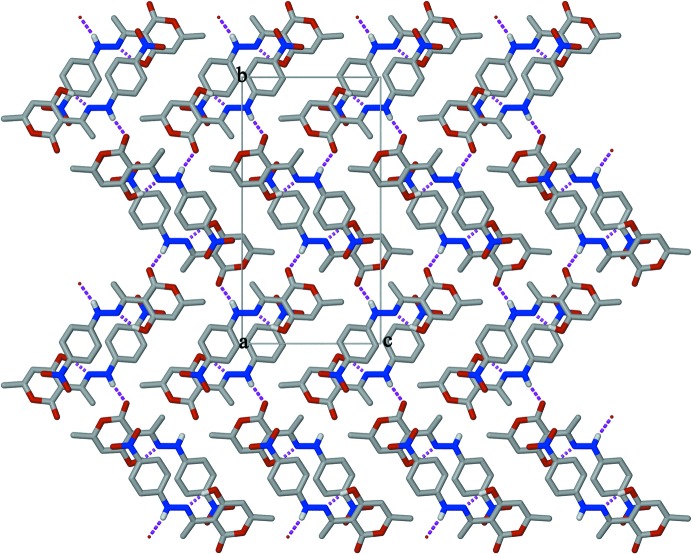
The crystal packing showing the herringbone arrangement of HMNP, viewed along the *a* axis. C-bound H atoms have been omitted for clarity. Hydrogen bonds are shown as dashed lines.

The crystal packing features strong N2—H2⋯O3^i^ hydrogen bonds between the NH group and the O_carbon­yl_ atom of the DHA moiety of symmetry-related mol­ecules, creating infinite chains along [101] (see Table 1 ▸ for symmetry code). This O_carbon­yl_ atom is also weakly hydrogen bonded to a symmetry-related hydrogen atom (C5–H5⋯O3^i^), forming a bifurcated N—H,C—H⋯O hydrogen bond (Fig. 2 ▸). In a similar fashion, the O2 atom of the pyran ring forms a weak hydrogen bond to the methyl hydrogen of an adjacent mol­ecule (C7—H7*A*⋯O2^i^). The chains are arranged in a herringbone pattern in the three-dimensional structure (Fig. 3 ▸).

## Hirshfeld surface analysis   

**Figure 4:**
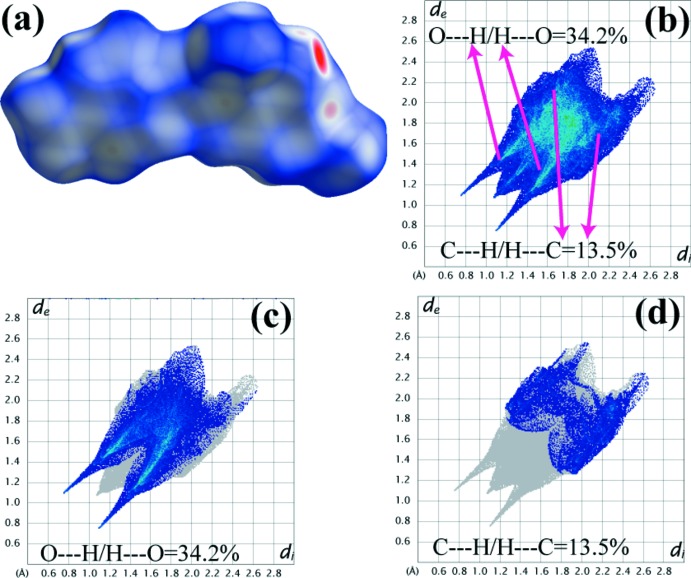
(*a*) Hirshfeld surfaces representation for HMNP mapped with *d*_norm_. (*b*)–(*d*) Fingerprint plots of HMNP resolved into different inter­molecular inter­actions showing the percentages of contacts contributing to the total Hirshfeld surface.

The Hirshfeld surface was mapped with *d*_norm_ to visualize the inter­molecular inter­actions and 2-D fingerprint plots were generated using *Crystal Explorer* (Wolff *et al.*, 2012 ▸) (Fig. 4 ▸).

## Spectroscopic and TG analysis   

The FT–IR spectrum of the title compound shows a characteristic peak at 1687 cm^−1^ which has been consigned for *ν*_C=N_, whereas the broad signal at 3280 cm^−1^ (*ν*_O–H_) indicates the presence of a phenolic group. The ^1^H NMR spectrum display a singlet at *δ* 15.23 ppm, which clearly indicates the dominance of the *enol* form of the title compound over the *keto* form. The absorption spectra for HMNP was recorded in C_2_H_5_OH, and λ_max_ was observed at 394 nm, which is ascribed to the π→π* or *n*→π* transition of the C=O or C=N group. To probe the thermal stability of HMNP, thermogravimetric analysis (TGA) was undertaken and it was found that HMNP is stable to 513 K.

## Synthesis and crystallization   

*Materials and methods: p*-Nitro­phenyl­hydrazine and de­hydro­acetic acid were of analytical grade and purchased from Spectrochem and Merck (India), respectively, and used as received. However, analytical grade solvents were purified wherever necessary as per as the standard literature method (Perrin *et al.*, 1980 ▸). The FT–IR spectra were recorded with a Perkin–Elmer FTIR–2000 spectrometer. The NMR spectroscopic measurements were carried out with a JEOL AL-400 MHz spectrometer. The thermogravimetric analysis (TGA) measurement was performed on an SDT Q600 (V20.9 Build 20) instrument (Artisan Technology Group, Champaign, IL) under N_2_ atmosphere with a heating rate of 10 K min^−1^. The absorbance spectrum was recorded on a JASCO V-530 UV/vis Spectrophotometer.

**Figure 5:**
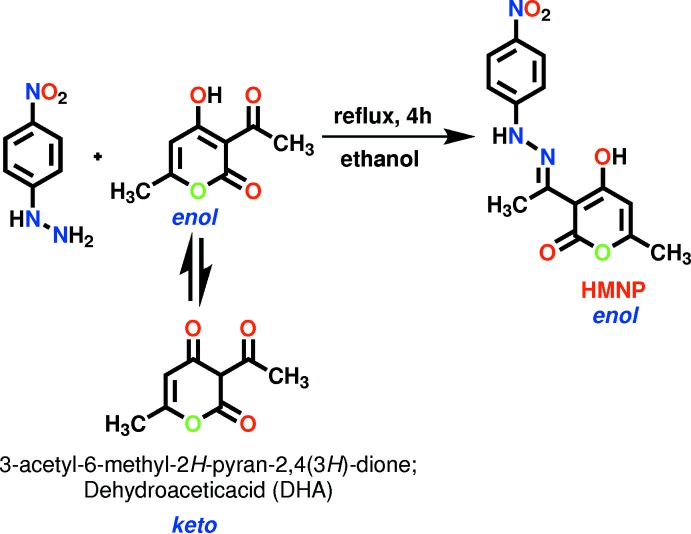
Synthetic route for the organic ligand HMNP.

*Synthesis of (E)-4-hy­droxy-6-methyl-3-(1-(2-(4-nitro­phen­yl) hydrazone) eth­yl) 2-H-pyran-2-one* (**HMNP**):

HMNP was synthesized by the reaction of DHA (0.56g, 0.003 mol) with *para*-nitro­phenyl­hydrazine (0.45g, 0.003 mol) in distilled ethanol (15 mL) under reflux condition at 353 K for 3 h (Fig. 5 ▸). The progress of the reaction was monitored by thin layer chromatography (TLC). After completion of the reaction, the reaction mixture was cooled to room temperature and the yellow crystalline precipitate was filtrated off and washed with cold ethanol and dried [yield: 0.728g (80%)]. Crystals suitable for single crystal X-ray analysis were obtained by the slow evaporation of a THF solution of HMNP for 7–8 d.

FT–IR (selected peaks): 3280 (O–H), 3088 (N–H), 1687 (C=O), 1646 (C=N) cm^−1^. Absorption spectrum [*λ*_max_, nm, C_2_H_5_OH (∊, *M* ^−1^ cm^−1^)]: 394 (150), 274 (*sh*, 525). ^1^H NMR (CDCl_3_, 400 MHz): *δ* (ppm): 15.23 (*s*, 1H, H_e_), 8.23–8.21 (*d*, 2H, H_a)_, 7.34 (1*s*, 1H, H_c_), 6.94–6.93 (*d*, 2H, H_b_), 5.93 (*s*, 1H, H_f_), 2.67 (1*s*, 3H, H_g_), 2.25 (1*s*, 3H, H_d_). ^13^C NMR (DMSO-*d_6_*, 100 MHz): *δ* 176.4 (C_8_), 167.1 (C_12_), 163.1 (C_10_), 150.2 (C_7_), 139.5 (C_4_), 125.8 (C_1_), 111.3 (C_2_), 103.3 (C_3_), 96.4 (C_9_), 79.1 (C_5_), 78.7 (C_11_), 78.3 (C_6_).

## Refinement   

Crystal data, data collection and structure refinement details are summarized in Table 2 ▸. The NH and OH hydrogen atoms were located in a difference-Fourier map and freely refined. The C-bound H atoms were included in calculated positions and treated as riding atoms: C—H = 0.93–0.96 Å, O—H= 0.82 Å with *U*_iso_(H) = 1.2*U*_eq_(C) and *U*_iso_(H) = 1.5*U*_eq_(C_meth­yl_). The crystal studied was a non-merohedral twin with the refined ratio of the twin components being 0.3720 (19):0.6280 (19) using twin matrix (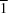
0 0) (0 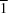 0) (0.265 0 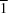).

## Supplementary Material

Crystal structure: contains datablock(s) I. DOI: 10.1107/S2056989017000639/lh5834sup1.cif

Structure factors: contains datablock(s) I. DOI: 10.1107/S2056989017000639/lh5834Isup2.hkl

Supporting information file. DOI: 10.1107/S2056989017000639/lh5834Isup3.cdx

Supporting information file. DOI: 10.1107/S2056989017000639/lh5834Isup4.cml

CCDC reference: 1515036

Additional supporting information:  crystallographic information; 3D view; checkCIF report

## Figures and Tables

**Table 1:** Hydrogen-bond geometry (Å, °)

*D*—H⋯*A*	*D*—H	H⋯*A*	*D*⋯*A*	*D*—H⋯*A*
O1—H1⋯N3	0.90 (2)	1.64 (2)	2.4760 (18)	154 (2)
N2—H2⋯O3^i^	0.85 (2)	2.00 (2)	2.8361 (19)	165.2 (19)
C5—H5⋯O3^i^	0.93	2.60	3.264 (2)	129
C7—H7*A*⋯O2^i^	0.96	2.51	3.283 (2)	138

Symmetry code: (i) 
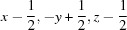
.

**Table 2:** Experimental details

Crystal data	
Chemical formula	C_14_H_13_N_3_O_5_
*M*_r_	303.27
Crystal system, space group	Monoclinic, *P*2_1_/*n*
Temperature (K)	297
*a*, *b*, *c* (Å)	6.9633 (3), 19.5008 (9), 10.2031 (5)
β (°)	95.196 (2)
*V* (Å^3^)	1379.78 (11)
*Z*	4
Radiation type	Mo *K*α
μ (mm^−1^)	0.11
Crystal size (mm)	0.16 × 0.13 × 0.10

Data collection	
Diffractometer	Bruker APEXII CCD
Absorption correction	Multi-scan (*TWINABS*; Sheldrick, 2012 ▸)
No. of measured, independent and observed [*I* > 2σ(*I*)] reflections	2696, 2696, 2302
*R*_int_	0.028
(sin θ/λ)_max_ (Å^−1^)	0.617

Refinement	
*R*[*F* ^2^ > 2σ(*F* ^2^)], *wR*(*F* ^2^), *S*	0.043, 0.121, 1.08
No. of reflections	2696
No. of parameters	208
No. of restraints	1
H-atom treatment	H atoms treated by a mixture of independent and constrained refinement
Δρ_max_, Δρ_min_ (e Å^−3^)	0.18, −0.20

Computer programs: *APEX2* and *SAINT* (Bruker, 2010 ▸), *SHELXS2013* (Sheldrick 2008 ▸), *SHELXL2016* (Sheldrick, 2015 ▸), *X-SEED* (Barbour 2001 ▸) and *publCIF* (Westrip 2010 ▸).

## supplementary crystallographic information

### Crystal data

**Table:**

C_14_H_13_N_3_O_5_	*F*(000) = 632
*M**_r_* = 303.27	*D*_x_ = 1.460 Mg m^−^^3^
Monoclinic, *P*2_1_/*n*	Mo *K*α radiation, λ = 0.71073 Å
*a* = 6.9633 (3) Å	Cell parameters from 9944 reflections
*b* = 19.5008 (9) Å	θ = 2.3–30.3°
*c* = 10.2031 (5) Å	µ = 0.11 mm^−^^1^
β = 95.196 (2)°	*T* = 297 K
*V* = 1379.78 (11) Å^3^	Block, colourless
*Z* = 4	0.16 × 0.13 × 0.10 mm

### Data collection

**Table:**

Bruker APEXII CCD diffractometer	2302 reflections with *I* > 2σ(*I*)
φ and ω scans	*R*_int_ = 0.028
Absorption correction: multi-scan (*TWINABS*; Sheldrick, 2012)	θ_max_ = 26.0°, θ_min_ = 2.3°
	*h* = −8→8
2696 measured reflections	*k* = 0→24
2696 independent reflections	*l* = 0→12

### Refinement

**Table:**

Refinement on *F*^2^	1 restraint
Least-squares matrix: full	Hydrogen site location: mixed
*R*[*F*^2^ > 2σ(*F*^2^)] = 0.043	H atoms treated by a mixture of independent and constrained refinement
*wR*(*F*^2^) = 0.121	*w* = 1/[σ^2^(*F*_o_^2^) + (0.0606*P*)^2^ + 0.3029*P*] where *P* = (*F*_o_^2^ + 2*F*_c_^2^)/3
*S* = 1.08	(Δ/σ)_max_ < 0.001
2696 reflections	Δρ_max_ = 0.18 e Å^−^^3^
208 parameters	Δρ_min_ = −0.20 e Å^−^^3^

### Special details

**Table:**

Geometry. All esds (except the esd in the dihedral angle between two l.s. planes) are estimated using the full covariance matrix. The cell esds are taken into account individually in the estimation of esds in distances, angles and torsion angles; correlations between esds in cell parameters are only used when they are defined by crystal symmetry. An approximate (isotropic) treatment of cell esds is used for estimating esds involving l.s. planes.
Refinement. Refined as a 2-component twin.

### Fractional atomic coordinates and isotropic or equivalent isotropic displacement parameters (Å^2^)

**Table:**

	*x*	*y*	*z*	*U*_iso_*/*U*_eq_	
O1	0.3744 (2)	0.45226 (6)	0.80648 (13)	0.0531 (4)	
H1	0.344 (4)	0.4379 (12)	0.7235 (19)	0.080*	
O2	0.6082 (2)	0.28918 (6)	1.00744 (12)	0.0469 (3)	
O3	0.6119 (3)	0.22490 (6)	0.83226 (13)	0.0663 (5)	
O4	0.0188 (3)	0.67218 (8)	0.2507 (2)	0.0812 (6)	
O5	−0.0004 (3)	0.61297 (9)	0.07175 (19)	0.0865 (6)	
N1	0.0349 (3)	0.61838 (9)	0.1917 (2)	0.0611 (5)	
N2	0.3120 (2)	0.38498 (7)	0.46572 (14)	0.0398 (4)	
H2	0.268 (3)	0.3474 (11)	0.432 (2)	0.048*	
N3	0.3581 (2)	0.38475 (6)	0.60024 (13)	0.0347 (3)	
C1	0.1015 (3)	0.55793 (9)	0.2667 (2)	0.0451 (5)	
C2	0.1693 (3)	0.56440 (9)	0.3967 (2)	0.0444 (4)	
H2A	0.167336	0.606928	0.437835	0.053*	
C3	0.2406 (3)	0.50781 (8)	0.46645 (17)	0.0391 (4)	
H3	0.289862	0.512366	0.553813	0.047*	
C4	0.2383 (2)	0.44367 (8)	0.40531 (16)	0.0337 (4)	
C5	0.1636 (3)	0.43827 (9)	0.27327 (18)	0.0462 (5)	
H5	0.158976	0.395663	0.232158	0.055*	
C6	0.0978 (3)	0.49483 (10)	0.20466 (19)	0.0517 (5)	
H6	0.050752	0.491001	0.116717	0.062*	
C7	0.5379 (3)	0.27794 (9)	0.57620 (17)	0.0454 (5)	
H7A	0.454884	0.238623	0.575635	0.068*	
H7B	0.664122	0.265763	0.614883	0.068*	
H7C	0.546326	0.293406	0.487576	0.068*	
C8	0.4574 (2)	0.33419 (8)	0.65505 (16)	0.0332 (4)	
C9	0.5723 (3)	0.28148 (8)	0.87201 (16)	0.0410 (4)	
C10	0.4942 (2)	0.33851 (8)	0.79792 (15)	0.0329 (4)	
C11	0.4516 (3)	0.39848 (8)	0.86652 (17)	0.0389 (4)	
C12	0.4968 (3)	0.40211 (10)	1.00539 (19)	0.0491 (5)	
H12	0.472308	0.442214	1.050290	0.059*	
C13	0.5736 (3)	0.34866 (9)	1.07078 (17)	0.0451 (4)	
C14	0.6316 (4)	0.34367 (13)	1.21487 (19)	0.0689 (7)	
H14A	0.564868	0.306098	1.251241	0.103*	
H14B	0.599050	0.385539	1.257136	0.103*	
H14C	0.768155	0.336166	1.228953	0.103*	

### Atomic displacement parameters (Å^2^)

**Table:**

	*U*^11^	*U*^22^	*U*^33^	*U*^12^	*U*^13^	*U*^23^
O1	0.0792 (10)	0.0393 (7)	0.0400 (7)	0.0220 (7)	0.0012 (7)	0.0015 (6)
O2	0.0651 (8)	0.0416 (7)	0.0322 (6)	0.0067 (6)	−0.0064 (6)	0.0044 (5)
O3	0.1158 (14)	0.0320 (6)	0.0454 (8)	0.0182 (8)	−0.0238 (8)	−0.0018 (6)
O4	0.0772 (12)	0.0448 (9)	0.1196 (16)	0.0129 (8)	−0.0011 (11)	0.0242 (9)
O5	0.0958 (14)	0.0791 (12)	0.0806 (13)	0.0013 (10)	−0.0138 (11)	0.0484 (10)
N1	0.0438 (9)	0.0498 (10)	0.0889 (15)	0.0011 (8)	0.0009 (9)	0.0340 (10)
N2	0.0555 (9)	0.0297 (7)	0.0317 (8)	−0.0015 (7)	−0.0101 (6)	0.0025 (6)
N3	0.0398 (8)	0.0327 (7)	0.0303 (7)	−0.0018 (6)	−0.0039 (6)	0.0047 (5)
C1	0.0382 (9)	0.0397 (9)	0.0563 (12)	−0.0006 (8)	−0.0016 (8)	0.0210 (8)
C2	0.0436 (10)	0.0312 (8)	0.0587 (12)	−0.0023 (8)	0.0059 (9)	0.0048 (8)
C3	0.0427 (9)	0.0347 (8)	0.0389 (10)	−0.0036 (7)	−0.0017 (8)	0.0025 (7)
C4	0.0349 (8)	0.0308 (8)	0.0345 (9)	−0.0030 (6)	−0.0025 (7)	0.0068 (6)
C5	0.0610 (11)	0.0383 (9)	0.0371 (10)	−0.0013 (9)	−0.0068 (9)	0.0042 (7)
C6	0.0611 (12)	0.0516 (11)	0.0396 (10)	−0.0018 (10)	−0.0102 (9)	0.0134 (8)
C7	0.0601 (12)	0.0401 (9)	0.0342 (9)	0.0088 (9)	−0.0052 (8)	−0.0031 (7)
C8	0.0364 (8)	0.0277 (7)	0.0344 (8)	−0.0031 (6)	−0.0026 (7)	0.0014 (6)
C9	0.0547 (11)	0.0331 (8)	0.0329 (9)	0.0011 (8)	−0.0083 (8)	0.0020 (7)
C10	0.0368 (8)	0.0301 (8)	0.0307 (8)	−0.0006 (6)	−0.0024 (7)	0.0023 (6)
C11	0.0458 (10)	0.0337 (8)	0.0371 (9)	0.0051 (7)	0.0028 (7)	0.0024 (7)
C12	0.0670 (13)	0.0450 (10)	0.0354 (9)	0.0098 (9)	0.0053 (9)	−0.0048 (8)
C13	0.0550 (11)	0.0494 (10)	0.0304 (9)	0.0041 (9)	0.0020 (8)	−0.0008 (8)
C14	0.0965 (18)	0.0774 (15)	0.0312 (10)	0.0156 (14)	−0.0026 (11)	−0.0018 (10)

### Geometric parameters (Å, º)

**Table:**

O1—C11	1.305 (2)	C4—C5	1.403 (2)
O1—H1	0.899 (17)	C5—C6	1.363 (2)
O2—C13	1.360 (2)	C5—H5	0.9300
O2—C9	1.390 (2)	C6—H6	0.9300
O3—C9	1.216 (2)	C7—C8	1.499 (2)
O4—N1	1.220 (2)	C7—H7A	0.9600
O5—N1	1.231 (3)	C7—H7B	0.9600
N1—C1	1.458 (2)	C7—H7C	0.9600
N2—C4	1.377 (2)	C8—C10	1.460 (2)
N2—N3	1.3809 (18)	C9—C10	1.424 (2)
N2—H2	0.85 (2)	C10—C11	1.408 (2)
N3—C8	1.301 (2)	C11—C12	1.425 (3)
C1—C2	1.373 (3)	C12—C13	1.324 (3)
C1—C6	1.383 (3)	C12—H12	0.9300
C2—C3	1.381 (2)	C13—C14	1.492 (2)
C2—H2A	0.9300	C14—H14A	0.9600
C3—C4	1.397 (2)	C14—H14B	0.9600
C3—H3	0.9300	C14—H14C	0.9600
			
C11—O1—H1	104.1 (16)	H7A—C7—H7B	109.5
C13—O2—C9	122.74 (13)	C8—C7—H7C	109.5
O4—N1—O5	123.05 (18)	H7A—C7—H7C	109.5
O4—N1—C1	118.4 (2)	H7B—C7—H7C	109.5
O5—N1—C1	118.5 (2)	N3—C8—C10	115.09 (14)
C4—N2—N3	119.41 (13)	N3—C8—C7	122.27 (14)
C4—N2—H2	115.4 (14)	C10—C8—C7	122.54 (14)
N3—N2—H2	116.0 (14)	O3—C9—O2	113.81 (14)
C8—N3—N2	119.75 (14)	O3—C9—C10	128.22 (15)
C2—C1—C6	120.96 (16)	O2—C9—C10	117.97 (14)
C2—C1—N1	119.85 (18)	C11—C10—C9	118.16 (15)
C6—C1—N1	119.18 (18)	C11—C10—C8	121.23 (14)
C1—C2—C3	120.03 (16)	C9—C10—C8	120.60 (14)
C1—C2—H2A	120.0	O1—C11—C10	122.02 (16)
C3—C2—H2A	120.0	O1—C11—C12	118.12 (15)
C2—C3—C4	119.77 (16)	C10—C11—C12	119.85 (15)
C2—C3—H3	120.1	C13—C12—C11	120.32 (17)
C4—C3—H3	120.1	C13—C12—H12	119.8
N2—C4—C3	123.76 (14)	C11—C12—H12	119.8
N2—C4—C5	117.25 (15)	C12—C13—O2	120.85 (16)
C3—C4—C5	118.95 (15)	C12—C13—C14	127.50 (18)
C6—C5—C4	120.68 (17)	O2—C13—C14	111.65 (16)
C6—C5—H5	119.7	C13—C14—H14A	109.5
C4—C5—H5	119.7	C13—C14—H14B	109.5
C5—C6—C1	119.57 (17)	H14A—C14—H14B	109.5
C5—C6—H6	120.2	C13—C14—H14C	109.5
C1—C6—H6	120.2	H14A—C14—H14C	109.5
C8—C7—H7A	109.5	H14B—C14—H14C	109.5
C8—C7—H7B	109.5		
			
C4—N2—N3—C8	−168.31 (16)	C13—O2—C9—C10	−0.6 (3)
O4—N1—C1—C2	−9.0 (3)	O3—C9—C10—C11	177.1 (2)
O5—N1—C1—C2	170.29 (19)	O2—C9—C10—C11	−2.2 (3)
O4—N1—C1—C6	172.0 (2)	O3—C9—C10—C8	−2.0 (3)
O5—N1—C1—C6	−8.7 (3)	O2—C9—C10—C8	178.60 (16)
C6—C1—C2—C3	1.9 (3)	N3—C8—C10—C11	−9.8 (2)
N1—C1—C2—C3	−177.08 (17)	C7—C8—C10—C11	166.71 (17)
C1—C2—C3—C4	−1.8 (3)	N3—C8—C10—C9	169.40 (16)
N3—N2—C4—C3	12.9 (3)	C7—C8—C10—C9	−14.1 (3)
N3—N2—C4—C5	−169.53 (16)	C9—C10—C11—O1	−177.92 (17)
C2—C3—C4—N2	177.75 (17)	C8—C10—C11—O1	1.3 (3)
C2—C3—C4—C5	0.2 (3)	C9—C10—C11—C12	3.5 (3)
N2—C4—C5—C6	−176.40 (19)	C8—C10—C11—C12	−177.33 (18)
C3—C4—C5—C6	1.3 (3)	O1—C11—C12—C13	179.38 (19)
C4—C5—C6—C1	−1.2 (3)	C10—C11—C12—C13	−2.0 (3)
C2—C1—C6—C5	−0.4 (3)	C11—C12—C13—O2	−0.9 (3)
N1—C1—C6—C5	178.59 (18)	C11—C12—C13—C14	179.3 (2)
N2—N3—C8—C10	−178.73 (15)	C9—O2—C13—C12	2.3 (3)
N2—N3—C8—C7	4.8 (2)	C9—O2—C13—C14	−177.92 (19)
C13—O2—C9—O3	179.91 (19)		

### Hydrogen-bond geometry (Å, º)

**Table:**

*D*—H···*A*	*D*—H	H···*A*	*D*···*A*	*D*—H···*A*
O1—H1···N3	0.90 (2)	1.64 (2)	2.4760 (18)	154 (2)
N2—H2···O3^i^	0.85 (2)	2.00 (2)	2.8361 (19)	165.2 (19)
C5—H5···O3^i^	0.93	2.60	3.264 (2)	129
C7—H7*A*···O2^i^	0.96	2.51	3.283 (2)	138

Symmetry code: (i) *x*−1/2, −*y*+1/2, *z*−1/2.
